# A novel approach with modified suture bridge fixation technique for posterior cruciate ligament tibial avulsion fracture in adult

**DOI:** 10.3389/fbioe.2025.1496728

**Published:** 2025-05-22

**Authors:** Xiong Wang, Qing Gu, Shuming Zi, Wenqiang Wei, Biao Cheng, Liehu Cao

**Affiliations:** ^1^ Department of Sports Medicine, Tongji Hospital, School of Medicine, Tongji University, Shanghai, China; ^2^ Department of Orthopedics, Shanghai Baoshan Luodian Hospital, Shanghai, China; ^3^ Department of Orthopedics, Shanghai Sixth People’s Hospital Affiliated to Shanghai Jiao Tong University School of Medicine, Shanghai, China

**Keywords:** posterior cruciate ligament, tibial avulsion fracture, suture bridge fixation, surgical treatment, outcome

## Abstract

**Background:**

Posterior cruciate ligament (PCL) tibial avulsion fractures are relatively rare injuries that often result in chronic pain, limited range of motion, knee instability, and osteoarthritis. Most cases require surgical intervention to restore the fragment’s normal anatomy, the ligament’s tension, and the knee joint’s stability. In this study, we propose a novel approach utilizing a modified suture bridge fixation technique to treat PCL tibial avulsion fractures and explore the clinical outcome and applicability.

**Methods:**

We retrospectively reviewed and collected the clinical data from March 2020 to April 2023. Of the 24 patients (14 males and 10 females) with PCL tibial avulsion fractures who underwent modified suture bridge fixation technique were enrolled in the study. The surgical data of the surgery time and intraoperative blood loss were analyzed. The knee range of motion (ROM), the Lysholm knee scoring scale, and the International Knee Documentation Committee (IKDC) were used to evaluate the recovery of knee joint function.

**Results:**

All 24 patients were followed up for a duration ranging from 11 to 16 months, with an average of 13.00 ± 1.32 months. The surgery time was 40∼60 min, with a mean of 50.88 ± 4.85 min. The intraoperative blood loss was approximately 25∼45 mL, averaging 36.75 ± 4.89 mL. No instances of wound infection, neurovascular injuries, fracture nonunion, fixation failure, deep vein thrombosis, or secondary operation were reported during follow-up. The knee joint range of motion (ROM) was 118°∼134°, with an average of 127.46° ± 4.16° at the final follow-up. The Lysholm score was 41.17 ± 3.48 at the preoperative stage and improved to 90.25 ± 2.59 at the final follow-up. The IKDC score was 40.38 ± 2.16 at the preoperative stage, and 88.54 ± 1.77 at the final follow-up.

**Conclusion:**

The results indicate that the novel approach utilizing a modified suture bridge fixation technique can provide effective stabilization and favorable clinical outcomes. The suture bridge structure can be applied to displaced posterior cruciate ligament (PCL) tibial avulsion fractures through its compression capabilities, especially in comminuted fractures. This procedure is straightforward, minimizing the risk of injury to peripheral neurovascular structures and eliminating the need for a second operation. Consequently, this technique represents a viable alternative treatment option for primary care facilities or hospitals that lack arthroscopic equipment.

## Introduction

Posterior cruciate ligament (PCL) injuries are relatively uncommon and frequently occur in young people due to traffic accidents or sports-related incidents, accounting for 3%–44% of all knee injuries (Hooper et al., 2018; Gopinatth et al., 2023). In contrast, PCL tibial avulsion fractures are relatively rare injuries; however, they often lead to chronic pain, instability, and the degeneration of osteoarthritis (Zhang et al., 2013; Ren et al., 2022). Surgical management is the most frequently employed treatment method (Sasaki et al., 2007). Restoring the anatomical position of the fragment, the tension of the ligament, and the stability of the knee joint are essential elements in the treatment of PCL tibial avulsion fractures (Jang and Lee, 2016).

Arthroscopic surgery has become more popular than ever before due to advances in science and technology. This procedure offers several advantages, including minimal trauma, rapid recovery, and the ability to simultaneously treat intra-articular concomitant injuries. However, the surgical procedures under arthroscopic are awkward for PCL tibial avulsion fractures due to their specific anatomical location on the posterior plateau and limited range of access. Arthroscopic surgery requires experienced sports medicine specialists and specialized equipment (Sabat et al., 2016; Zhu et al., 2017). Additionally, mastering this technique involves a long learning curve. Furthermore, fresh PCL tibial avulsion fractures often present with significant bleeding, which may impair arthroscopic visibility. Open surgery provides direct access to the fracture site, allowing for anatomical reduction and rigid fixation of fragments (Ambra et al., 2016). More crucially, open surgery is particularly suitable for primary care facilities, particularly those lacking arthroscopic equipment and experience in arthroscopic procedures. Research has demonstrated the efficacy of utilizing screws, sutures, anchors, Endobutton, and plates in achieving favorable outcomes (Li et al., 2018; Deng et al., 2020). Nevertheless, the inherent challenges associated with conventional internal fixation in open surgery are considerable, including the potential for damage to the neurovascular structure, scarring of the wound, iatrogenic fracture, internal fixation failure, and the necessity for a secondary operation to remove internal devices (Abdallah and Arafa, 2017). Consequently, proposing a novel surgical approach and fixation method for the treatment of PCL tibial avulsion fractures could provide a more effective means of reducing postoperative complications and promoting the rehabilitation of knee function.

The suture bridge fixation technique has been successfully employed in the treatment of ripped rotator cuffs and anterior cruciate ligament tibial avulsion fractures with suture anchors, resulting in satisfactory recovery after the operation (Hantes et al., 2018; Wang et al., 2024). In this study, we put forward a novel approach with a modified suture bridge fixation technique to reduce and stabilize PCL tibial avulsion fractures. The middle portion of the medial head of the gastrocnemius was bluntly separated to expose the posterior articular compartment through a posterior arc-shaped approach. The avulsion fracture fragment was squeezed with four sutures that traversed from the base of PCL, originating from a Suture anchor situated at the superior edge of the bone bed. These sutures were loaded with a Knotless anchor on the inferior edge of the bone bed.

This study aimed to evaluate the efficacy of open surgery utilizing a novel approach with a modified suture bridge fixation technique in the treatment of PCL tibial avulsion fractures. We hypothesized that this modified surgical technique could reduce the risk of neurovascular bundle injury, facilitate direct observation and fixation of the fracture fragment, avoid the necessity for a secondary operation, and permit early functional rehabilitation and return to work.

## Materials and methods

### Patients

**FIGURE 1:**
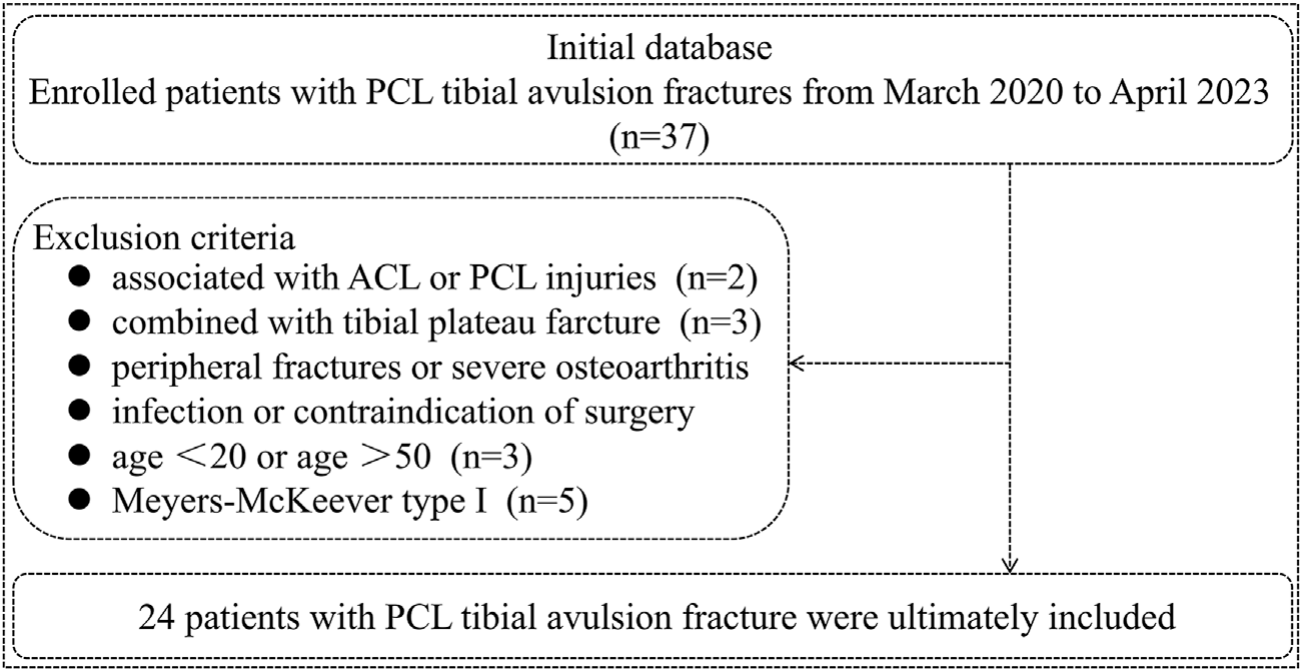
The schematic diagram illustrates the case inclusion and exclusion process.

A retrospective analysis was conducted on 37 cases of PCL tibial avulsion fractures from March 2020 to April 2023. The inclusion criteria for the enrolled patients are as follows. (1) Fresh PCL tibial avulsion fracture (occurring within 2 weeks); (2) Age between 20 and 50 years; (3) Imaging examination before surgery (X-ray, CT, or MRI); (4) Meyers-McKeever type II and III fracture; (5) The injured knee exhibited dysfunction in flexion and extension; (6) Agreement to participate in this study and sign informed consent; (7) Follow-up for at least 11 months and completion of relevant examinations. Exclusion criteria include ACL or PCL injury, peripheral fractures, severe osteoarthritis, knee joint infection, and contraindications to surgery. Based on the inclusion and exclusion criteria, 24 patients were ultimately included in this study (Figure 1). All patients underwent a novel approach with a modified suture bridge fixation method. We obtained the informed consent of all patients before the operation. Furthermore, this study was approved by our institutional review board.

### Operation procedure

**FIGURE 2:**
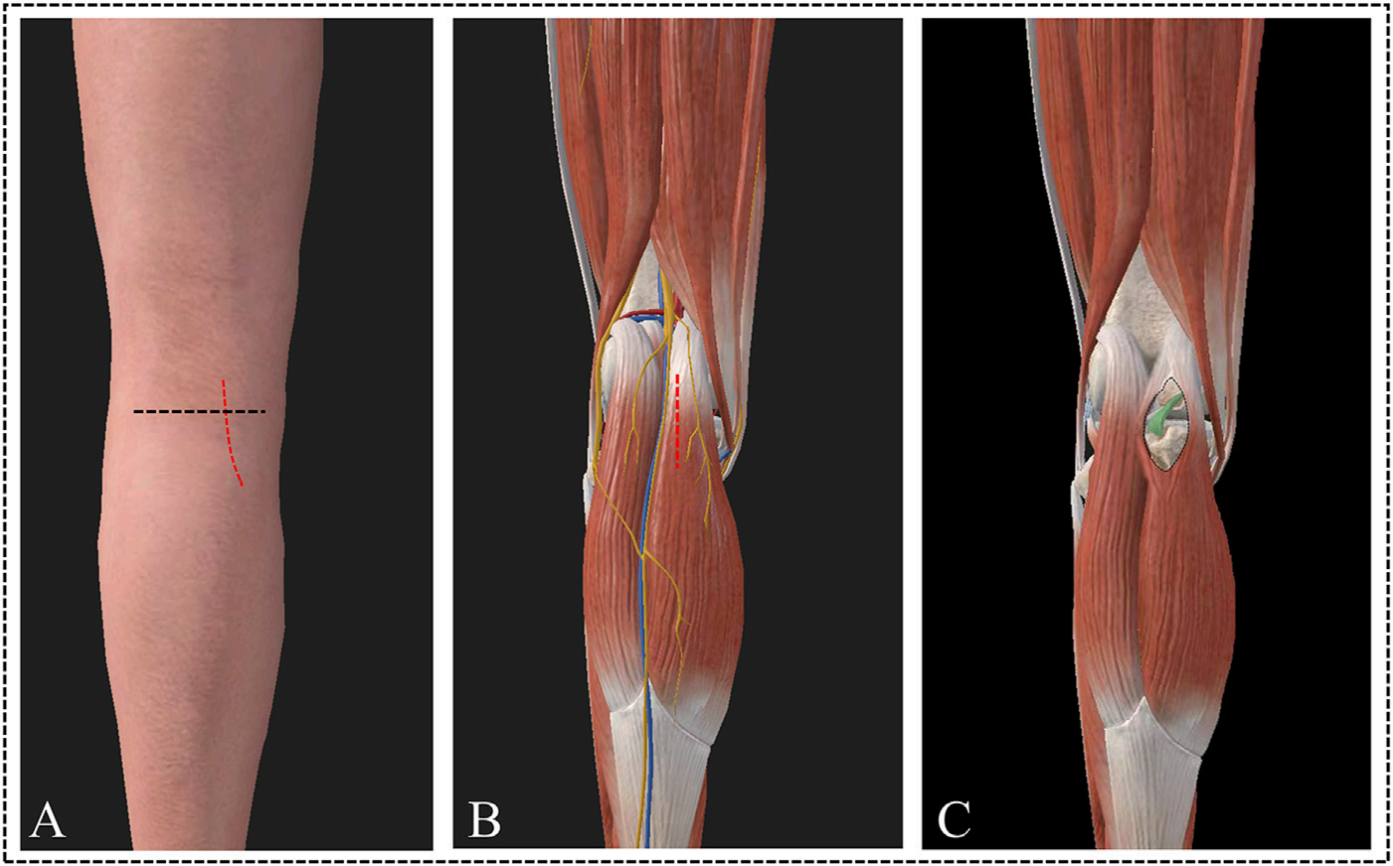
A novel approach for PCL tibial avulsion fracture **(A)** shows an arc-shaped incision originating from the popliteal upper border and extending outward and downward. **(B)** Shows the incision made on the middle of the medial head of the gastrocnemius. **(C)** Shows the approach used to expose the fracture region.

**FIGURE 3:**
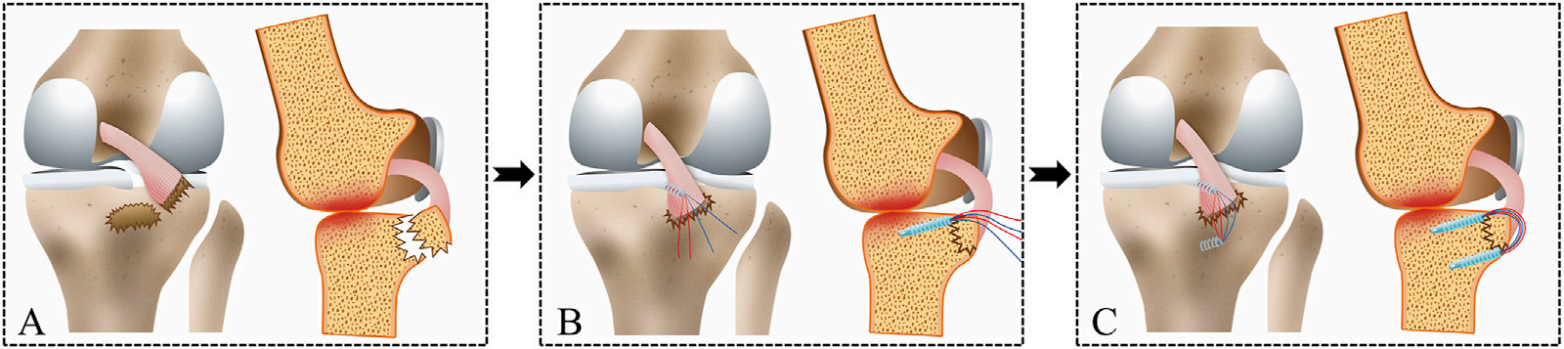
The following diagram illustrates the operation of the modified suture bridge fixation technique for a PCL tibial avulsion fracture. **(A)** PCL tibial avulsion fracture. **(B)** A Suture anchor was inserted into the superior edge of the bone bed, and the sutures were passed through PCL. **(C)** The distributed sutures from the Suture anchor were loaded with a Knotless anchor on the inferior edge of the bone bed.

After successful anesthesia, the patient was positioned in the prone position, and an electric pneumatic tourniquet was applied. The knee joint was flexed from 30° to 45° to fully relax the medial gastrocnemius muscle and the surrounding tissues. After thorough sterilization of the surgical area, it was covered with a sterile membrane and sheets. The approach employs an arc-shaped incision that commences at the upper border of the popliteal striae along the medial margin of the gastrocnemius muscle and extends outward and downward. Subsequently, the middle portion of the medial head of the gastrocnemius is bluntly separated into two parts. The two parts of the separated gastrocnemius muscle could then be stretched, allowing for exposure of the posterior joint capsule. Once the posterior joint capsule was opened, the attachment site of PCL and operation space were exposed (Figure 2). Additionally, a diagram of the modified suture bridge fixation technique for PCL tibial avulsion fractures is provided to facilitate a comprehensive understanding of the procedures (Figure 3).

**FIGURE 4:**
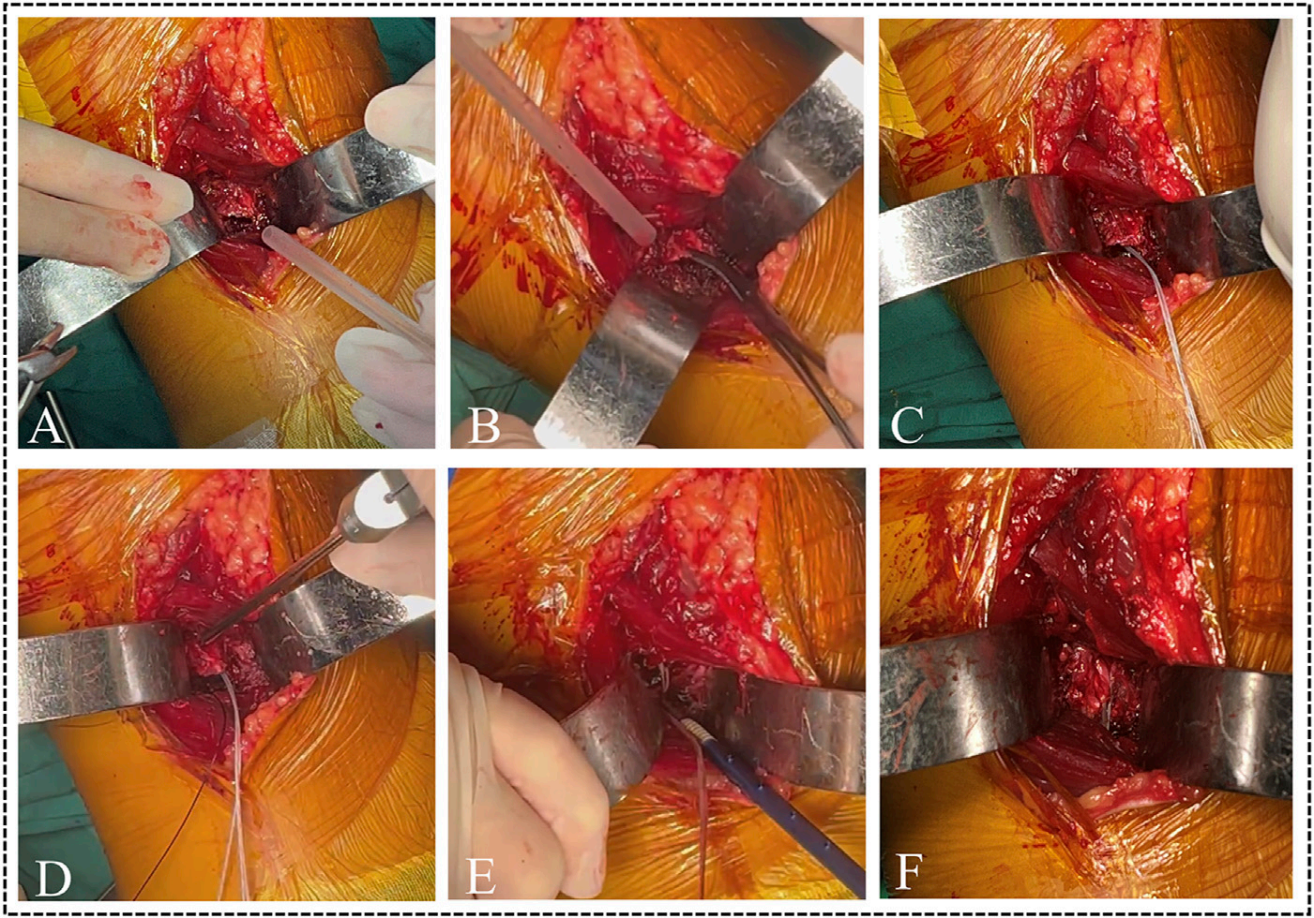
The intraoperative images of the modified suture bridge fixation technique. **(A)** Exposing the PCL tibial avulsion fracture site. **(B)** Elevating the fragment and cleaning the blood and damaged soft tissue. **(C,D)** Inserting a suture anchor and passing sutures to the bases of PCL. **(E,F)** Constructing a suture bridge structure utilizing a knotless anchor.

The fracture fragment was elevated, and the blood and compressed soft tissue under the bone bed were cleaned and washed. Subsequently, an absorbable Suture anchor was inserted into the superior edge of the bone bed. Four sutures were passed through the base of the PCL using the suture hook with PDS II. The fracture was reduced and could be stabilized using a 1.0 mm Kirschner wire. A C-arm machine equipped with a sterile device cover was utilized to assess the quality of reduction from both anterior-posterior and lateral views. After that, a Knotless anchor penetrated the inferior edge of the bone bed. The four sutures from the Suture anchor were loaded with Knotless anchor to form a suture bridge structure, which compresses and maintains the reduction of the avulsion fracture (Figure 4). Finally, the stability of the avulsion fracture was assessed through active knee flexion and extension. After achieving complete hemostasis, the surgical region was irrigated and sutured layer by layer.

### Postoperative management

Prophylactic cefuroxime sodium (1.5 g) was administered half an hour preoperatively and continued for 24 h postoperatively to prevent infection. An adjustable knee brace was used to immobilize the knee joint within 30° immediately after surgery. In addition, rehabilitation exercises were recommended to increase muscle strength and prevent deep vein thrombosis, such as isometric quadriceps, ankle pump exercises, and straight leg raising. During the first 2 weeks, the adjustable brace could be gradually adjusted to 60° depending on the conditions of functional recovery. From two to 4 weeks, the adjustable brace was adjusted to 90°, and the injured leg was allowed to toe contact or no weight-bearing walking. At 6 weeks, the adjustable brace was adjusted to 120°, and partial-weight bearing was allowed. The adjustable brace could be removed after 8 weeks. When X-ray showed the fracture healing, full weight-bearing walking and activities of daily life were permitted.

Baseline information and surgical data for these patients were collected and analyzed from the Electronic Medical Records System. X-rays, CT scans, or MRI scans were obtained to assess fracture union conditions during follow-up. The follow-up time, surgery time, and intraoperative blood loss were recorded. The knee range of motion (ROM), the Lysholm knee scoring scale, and the International Knee Documentation Committee (IKDC) were used to assess knee functional recovery.

## Statistical analysis

Statistical analyses were performed using SPSS software version 25.0 (SPSS, Chicago, IL, United States). Clinical data with a normal distribution were presented as mean ± standard deviation. A paired t-test was used to compare the IKDC and Lysholm scores between the preoperative and final follow-up. The difference was statistically significant with P < 0.05.

## Results

Of the 24 patients (14 males, 10 females), the average age was 37.37 years (22∼48 years). The most common mechanisms of injury included motorcycle accidents (13 patients), sports-related injuries (6 patients), and falls from heights (5 patients). According to the Meyers-McKeever classification system, which is based on the extent of the fragment displacement observed on radiographs, 10 patients were classified as type II, and 14 patients as type III (Table 1).

**TABLE 1:** General characteristics of the patients (n = 24).

Parameter	Results
Number of male: female patient	14 : 10
Age (years)	37.37 (22∼48)
Injury mechanism	
Motorcycle accident	13 (54.2%)
Sports injury	6 (25.0%)
Fall from heights	5 (20.8%)
Meyers-McKeever classification	
Type II	10 (41.7%)
Type III	14 (58.3%)
Surgical data	
Follow-up (months)	13.00 ± 1.32 (11∼16)
Surgery time (min)	50.88 ± 4.85 (40∼60)
The intraoperative blood loss (mL)	36.75 ± 4.89 (25∼45)
Knee joint range of motion (°)	127.46 ± 4.16 (118∼134)

All 24 patients were followed up for 11∼16 months with a mean of 13.00 ± 1.32 months. The surgery time was 40∼60 min, with a mean of 50.88 ± 4.85 min. The intraoperative blood loss was approximately 25∼45 mL, with a mean of 36.75 ± 4.89 mL. There were no instances of wound infection, neurovascular injuries, fracture nonunion, fixation failure, deep vein thrombosis, or secondary operation during the follow-up period.

**FIGURE 5:**
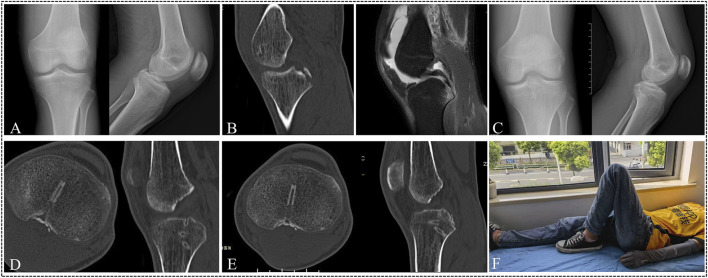
A 26-year-old male suffered a displaced PCL tibial avulsion fracture of the left knee as a result of motorcycle traffic. **(A–C)** The X-ray, CT, and MRI images demonstrated the presence of a PCL tibial avulsion fracture. **(D)** The X-ray images showed fracture fragment reduction well at 2 weeks after operation. **(E)** The CT images revealed that there was no evidence of fracture displacement at 6 weeks after the operation. **(F)** At 10 weeks, the CT image demonstrated evidence of fracture union. G At 12 weeks, the patient was satisfied with the surgical outcome and returned to previous work.

At the final follow-up, the knee joint range of motion (ROM) was 118°∼134°, with an average of 127.46° ± 4.16°. The Lysholm score was 41.17 ± 3.48 preoperatively and 90.25 ± 2.59 at the final follow-up. The IKDC score was 40.38 ± 2.16 preoperatively and 88.54 ± 1.77 at the final follow-up (Table 2). All patients received adequate postoperative rehabilitation after the operation. They were satisfied with the surgical outcomes and successfully returned to their normal work and daily activities (Figure 5).

**TABLE 2:** Comparison of preoperative and final follow-up of knee function conditions.

Parameter	Preoperative	Final follow-up	T-value	P-value
Lysholm	41.17 ± 3.48	90.25 ± 2.59	−62.556	0.000
IKDC	40.38 ± 2.16	88.54 ± 1.77	−89.095	0.000

## Discussion

The primary function of the intact posterior cruciate ligament (PCL) is to restrain posterior tibial translation and maintain knee joint stability. Injury to the tibial insertion of the PCL is a special damage, and the incident rate is relatively uncommon (Green et al., 2019; Hassebrock et al., 2020). The most common symptoms include high articular cartilage pressure, instability of the knee joint, limited range of motion, and impaired quality of life. Effective treatment is beneficial in restoring the stability of the knee joint, improving knee joint function, and facilitating a timely return to normal activities.

The existing literature demonstrates that arthroscopic and open surgical interventions are superior to conservative treatment for PCL tibial avulsion fractures, particularly for mildly displaced “hinged” fractures (type II) and completely detached avulsion fractures (type III) (Griffith et al., 2004). Whereas the choice of surgical method is determined by the size of the fracture fragments. For small fragments less than 10 mm, suture or anchor fixation is deemed appropriate. For fragment sizes between 10 mm and 20 mm, the possible options include screw, Kirschner wire, or suture anchor fixation. For fragments larger than 20 mm, screw, plate, or suture anchor fixation is considered suitable. However, complications such as an iatrogenic fracture, reduction loosening, or intercondylar impingement may occur with screw or Kirschner wire fixation (Keyhani et al., 2022). Conversely, plate fixation may result in chronic soreness and irritation, and some of them may necessitate a secondary operation to remove internal fixation (Kim et al., 2001).

The standard approach for PCL avulsion fracture is the retrogenicular inverted “L” approach, initially described by Burcks and Schaffer (Burks and Schaffer, 1990). The fracture region is accessible through the interspace between the medial head of the gastrocnemius and the semimembranosus muscle. However, the incision is relatively large, which increases the risk of damage to the surrounding blood vessels and nerves. The “S-shaped” approach was proposed to minimize traction on the gastrocnemius muscle during the surgical procedure. However, this approach did not effectively reduce damage to the neurovascular bundle or simplify the surgical procedure (Nicandri et al., 2008). The direct lateral posterior approach was employed to identify the fracture region through the longitudinal splitting of the lateral head of the gastrocnemius muscle. Nevertheless, this approach is infrequently utilized in practice, and its therapeutic efficacy remains uncertain in clinics (Keyhani et al., 2022). The interval between the medial and lateral heads of the gastrocnemius in the posterior approach allows for direct exposure of the operation region. However, this approach is associated with an increased risk of iatrogenic injury to surrounding tissue (Khalifa et al., 2021). It is therefore crucial to adopt an effective approach that minimizes the risk of injury to the neurovascular bundle and ensures a simple operation to effectively manage PCL tibial avulsion fractures. In this study, a novel posterior arc-incision approach was adopted. The middle portion of the medial head of the gastrocnemius muscle was bluntly separated and retracted to both sides, thereby exposing the surgical region. This approach effectively avoids damage to surrounding tissue and streamlines the surgical procedure. The incision healed well, with no instances of wound infection, neurovascular injuries, or deep vein thrombosis during follow-up.

The average age of enrolled patients was 37.37 years (22∼48 years). These patients represent the primary labor force and breadwinners of their families. Therefore, they need to restore stability and function to the knee while minimizing postoperative complications. A review of the literature reveals that suture bridge fixation exhibits higher failure loads and fixation strength than other fixation techniques (Hapa et al., 2012; Schneppendahl et al., 2012). Compared to screw or plate fixations reported in the literature, our novel approach utilizing a modified suture bridge fixation technique with absorbable anchors results in minimal intraoperative blood loss and reduced operative time, achieving better prognostic outcomes without a secondary operation to remove the internal fixation (Wang et al., 2024). In addition, the suture bridge structure can provide sufficient stability for comminuted avulsion fractures via compression capabilities. The mean surgical time was 50.88 ± 4.85 min, and the mean intraoperative blood loss was 36.75 ± 4.89 mL. The mean knee joint range of motion at the final follow-up was 127.46° ± 4.16°, with a range of 118°–134°. At the final follow-up, the Lysholm score was 90.25 ± 2.59; while the IKDC score was 88.54 ± 1.77. The functional recovery observed in this study was comparable to that reported in a previous study that employed a minimally invasive approach with screw fixation for the treatment of PCL avulsion fractures (Guo et al., 2023). In that study, the average surgical time was 54.46 ± 7.64 min, the average blood loss was 48.85 ± 5.88 mL, the Lysholm score was 94.12 ± 2.49, and the IKDC score was 91.85 ± 2.19. Deng et al. confirmed that the hook plate could obtain satisfactory outcomes and reliable fixation in PCL tibial avulsion fractures, with a knee joint range of motion of 127.75° ± 6.13°, and an LKSS score of 92.75 ± 5.46. However, this method involved a large incision, a higher risk of injury to peripheral neurovascular structures, great trauma, and higher postoperative complications such as secondary operation (Deng et al., 2020; Liu et al., 2021).

The limitations of this study are listed as follows. Firstly, this study is a retrospective study with a relatively small number of cases. Secondly. The study lacks a control group utilizing alternative methods, such as screw and plate fixation. Thirdly, with the increasing incidence of traffic accidents and an aging population, individuals from various age groups may experience PCL tibial avulsion fractures. Therefore, further analysis involving diverse age groups, large sample sizes, and multicenter randomized controlled trials should be conducted. Moreover, a biomechanical study is required to elucidate the relative merits and limitations of suture bridge fixation compared to other fixation methods.

## Conclusion

A novel approach with a modified suture bridge fixation technique can yield favorable clinical outcomes in treating displaced and comminuted posterior cruciate ligament (PCL) tibial avulsion fractures through its compression capabilities. This straightforward surgical procedure can minimize the risk of injury to peripheral neurovascular structures and eliminate the necessity for a secondary operation. It serves as a viable alternative treatment option for primary care facilities or hospitals that lack the necessary arthroscopic equipment.

## Data Availability

The original contributions presented in the study are included in the article/supplementary material, further inquiries can be directed to the corresponding author.

## References

[B1] Abdallah A. A. Arafa M. S. (2017). Treatment of posterior cruciate ligament tibial avulsion by a minimally-invasive open posterior approach. Injury-International J. Care Inj. 48, 1644–1649. 10.1016/j.injury.2017.05.032 28577891

[B2] Ambra L. F. M. Franciozi C. E. S. Werneck L. G. M. De Queiroz A. a.B. Yamada R. K. Granata G. S. M. (2016). Posteromedial versus direct posterior approach for posterior cruciate ligament reinsertion. Orthopedics 39, E1024–E1027. 10.3928/01477447-20160623-15 27398782

[B3] Burks R. T. Schaffer J. J. (1990). A simplified approach to the tibial attachment of the posterior cruciate ligament. Clin. Orthop. Relat. Res. 254, 216–219. 10.1097/00003086-199005000-00031 2323134

[B4] Deng W. Li Y. X. Wu S. Z. Liu X. Huang F. G. Zhang H. (2020). Surgical treatment of posterior cruciate ligament tibial avulsion fractures using a locking compression hook plate: a case series. Acta Orthop. Traumatologica Turcica 54, 623–626. 10.5152/j.aott.2020.19244 PMC7815219 33423996

[B5] Gopinatth V. Mameri E. S. Casanova F. J. Khan Z. A. Jackson G. R. Mccormick J. R. (2023). Systematic review and meta-analysis of clinical outcomes after management of posterior cruciate ligament tibial avulsion fractures. Orthop. J. Sports Med. 11, 23259671231188383. 10.1177/23259671231188383 37724253 PMC10505349

[B6] Green D. Tuca M. Luderowski E. Gausden E. Goodbody C. Konin G. (2019). A new, MRI-based classification system for tibial spine fractures changes clinical treatment recommendations when compared to Myers and Mckeever. Knee Surg. Sports Traumatol. Arthrosc. 27, 86–92. 10.1007/s00167-018-5039-7 29961096

[B7] Griffith J. F. Antonio G. E. Tong C. W. C. Ming C. K. (2004). Cruciate ligament avulsion fractures. Arthroscopy-the J. Arthrosc. Relat. Surg. 20, 803–812. 10.1016/j.arthro.2004.06.007 15483540

[B8] Guo H. H. Zhao Y. Gao L. Wang C. Shang X. B. Fan H. T. (2023). Treatment of avulsion fracture of posterior cruciate ligament tibial insertion by minimally invasive approach in posterior medial knee. Front. Surg. 9, 885669. 10.3389/fsurg.2022.885669 36684149 PMC9852621

[B9] Hantes M. E. Ono Y. Raoulis V. A. Doxariotis N. Venouziou A. Zibis A. (2018). Arthroscopic single-row versus double-row suture bridge technique for rotator cuff tears in patients younger than 55 Years: a prospective comparative study. Am. J. Sports Med. 46, 116–121. 10.1177/0363546517728718 28942685

[B10] Hapa O. Barber F. A. Süner G. Özden R. Davul S. Bozdag E. (2012). Biomechanical comparison of tibial eminence fracture fixation with high-strength suture, EndoButton, and suture anchor. Arthroscopy-the J. Arthrosc. Relat. Surg. 28, 681–687. 10.1016/j.arthro.2011.10.026 22284410

[B11] Hassebrock J. D. Gulbrandsen M. T. Asprey W. L. Makovicka J. L. Chhabra A. (2020). Knee ligament anatomy and biomechanics. Sports Med. Arthrosc. Rev. 28, 80–86. 10.1097/jsa.0000000000000279 32740458

[B12] Hooper P. O. Silko C. Malcolm T. L. Farrow L. D. (2018). Management of Posterior Cruciate Ligament Tibial Avulsion Injuries: A Systematic Review. Am J Sports Med 46, 734–742. 10.1177/0363546517701911 28437619

[B13] Jang K. M. Lee S. H. (2016). Delayed surgical treatment for tibial avulsion fracture of the posterior cruciate ligament in children. Knee Surg. Sports Traumatol. Arthrosc. 24, 754–759. 10.1007/s00167-015-3929-5 26704790

[B14] Keyhani S. Soleymanha M. Salari A. (2022). Treatment of posterior cruciate ligament tibial avulsion: a new modified open direct lateral posterior approach. J. Knee Surg. 35, 862–867. 10.1055/s-0040-1721093 33241541

[B15] Khalifa A. A. Elsherif M. E. Elsherif E. Refai O. (2021). Posterior cruciate ligament tibial insertion avulsion, management by open reduction and internal fixation using plate and screws through a direct posterior approach. Injury-International J. Care Inj. 52, 594–601. 10.1016/j.injury.2020.09.058 33023741

[B16] Kim S. J. Shin S. J. Choi N. H. Cho S. K. (2001). Arthroscopically assisted treatment of avulsion fractures of the posterior cruciate ligament from the tibia. J. Bone Jt. Surgery-American 83A, 698–708. 10.2106/00004623-200105000-00008 11379739

[B17] Li J. Yu Y. Liu C. H. Su X. Z. Liao W. X. Li Z. L. (2018). Arthroscopic fixation of tibial eminence fractures: a biomechanical comparative study of screw, suture, and suture anchor. Arthroscopy-the J. Arthrosc. Relat. Surg. 34, 1608–1616. 10.1016/j.arthro.2017.12.018 29397286

[B18] Liu H. Liu J. Wu Y. W. Ma Y. H. Gu S. J. Mi J. Y. (2021). Outcomes of tibial avulsion fracture of the posterior cruciate ligament treated with a homemade hook plate. Injury-International J. Care Inj. 52, 1934–1938. 10.1016/j.injury.2021.04.042 33934882

[B19] Nicandri G. T. Klineherg E. O. Wahl C. J. Mills W. J. (2008). Treatment of posterior cruciate ligament tibial avulsion fractures through a modified open posterior approach: operative technique and 12-to 48-month outcomes. J. Orthop. Trauma 22, 317–324. 10.1097/bot.0b013e31817279d1 18448985

[B20] Ren X. Y. Wang J. N. Yang S. L. Liu Z. Wang T. D. Zhang T. (2022). The safety, efficacy, and functional outcomes on arthroscopic fixation of posterior cruciate ligament avulsion fracture by a bio-absorbable anchor or traditional pull-out technique: a prospective cohort study. Front. Bioeng. Biotechnol. 10, 1055176. 10.3389/fbioe.2022.1055176 36466345 PMC9708702

[B21] Sabat D. Jain A. Kumar V. (2016). Displaced posterior cruciate ligament avulsion fractures: a retrospective comparative study between open posterior approach and arthroscopic single-tunnel suture fixation. Arthroscopy-the J. Arthrosc. Relat. Surg. 32, 44–53. 10.1016/j.arthro.2015.06.014 26311286

[B22] Sasaki S. U. Albuquerque R. Amatuzzi M. M. Pereira C. a.M. (2007). Open screw fixation versus Arthroscopic suture fixation of tibial posterior cruciate ligament avulsion injuries: a mechanical comparison. Arthroscopy-the J. Arthrosc. Relat. Surg. 23, 1226–1230. 10.1016/j.arthro.2007.06.012 17986411

[B23] Schneppendahl J. Thelen S. Gehrmann S. Twehues S. Eichler C. Koebke J. (2012). Biomechanical stability of different suture fixation techniques for tibial eminence fractures. Knee Surg. Sports Traumatol. Arthrosc. 20, 2092–2097. 10.1007/s00167-011-1838-9 22203047

[B24] Wang X. Zi S. M. Ji X. X. Zhu W. H. Cao L. H. (2024). A novel approach for anterior cruciate ligament tibial avulsion fracture: arthroscopic modified suture bridge fixation technique. Archives Orthop. Trauma Surg. 144, 3167–3173. 10.1007/s00402-024-05365-8 38904681

[B25] Zhang X. C. Cai G. P. Xu J. Wang K. (2013). A minimally invasive postero-medial approach with suture anchors for isolated tibial avulsion fracture of the posterior cruciate ligament. Knee 20, 96–99. 10.1016/j.knee.2012.10.016 23159153

[B26] Zhu W. Lu W. Cui J. Peng L. Ou Y. Li H. (2017). Treatment of tibia avulsion fracture of posterior cruciate ligament with high-strength suture fixation under arthroscopy. Eur. J. Trauma Emerg. Surg. 43, 137–143. 10.1007/s00068-015-0606-9 26660676 PMC5306319

